# Modeling Electric Fields in Transcutaneous Spinal Direct Current Stimulation: A Clinical Perspective

**DOI:** 10.3390/biomedicines11051283

**Published:** 2023-04-26

**Authors:** Matteo Guidetti, Stefano Giannoni-Luza, Tommaso Bocci, Kevin Pacheco-Barrios, Anna Maria Bianchi, Marta Parazzini, Silvio Ionta, Roberta Ferrucci, Natale Vincenzo Maiorana, Federico Verde, Nicola Ticozzi, Vincenzo Silani, Alberto Priori

**Affiliations:** 1 Aldo Ravelli Research Center for Neurotechnology and Experimental Neurotherapeutics, Department of Health Sciences, University of Milan, 20142 Milan, Italy; matteo.guidetti@unimi.it (M.G.); tommaso.bocci@unimi.it (T.B.); natale.maiorana@unimi.it (N.V.M.); 2 Department of Electronics, Information and Bioengineering, Politecnico di Milano, 20133 Milan, Italy; annamaria.bianchi@polimi.it; 3 Sensory-Motor Lab (SeMoLa), Department of Ophthalmology—University of Lausanne, Jules Gonin Eye Hospital/Fondation Asile des Aveugles, 1015 Lausanne, Switzerland; sgiannonil@gmail.com (S.G.-L.); ionta.silvio@gmail.com (S.I.); 4 III Neurology Clinic, ASST-Santi Paolo e Carlo University Hospital, 20142 Milan, Italy; roberta.ferrucci@unimi.it; 5 Neuromodulation Center and Center for Clinical Research Learning, Spaulding Rehabilitation Hospital and Massachusetts General Hospital, Boston, MA 02129, USA; kevin.pacheco.barrios@gmail.com; 6 Unidad de Investigación para la Generación y Síntesis de Evidencias en Salud, Universidad San Ignacio de Loyola, Vicerrectorado de Investigación, Lima 15024, Peru; 7 Istituto di Elettronica e di Ingegneria Dell’Informazione e delle Telecomunicazioni (IEIIT), Consiglio Nazionale delle Ricerche (CNR), 10129 Milan, Italy; marta.parazzini@ieiit.cnr.it; 8 Department of Oncology and Hematology, University of Milan, 20122 Milan, Italy; 9 Department of Neurology, Istituto Auxologico Italiano IRCCS, 20149 Milan, Italy; federico.verde@unimi.it (F.V.); nicola.ticozzi@unimi.it (N.T.); vincenzo.silani@unimi.it (V.S.); 10 Department of Pathophysiology and Transplantation, ‘Dino Ferrari’ Center, Università degli Studi di Milano, 20122 Milan, Italy

**Keywords:** non-invasive brain stimulation, neuromodulation, transcutaneous spinal direct current stimulation, electric fields, computational models, clinical study

## Abstract

Clinical findings suggest that transcutaneous spinal direct current stimulation (tsDCS) can modulate ascending sensitive, descending corticospinal, and segmental pathways in the spinal cord (SC). However, several aspects of the stimulation have not been completely understood, and realistic computational models based on MRI are the gold standard to predict the interaction between tsDCS-induced electric fields and anatomy. Here, we review the electric fields distribution in the SC during tsDCS as predicted by MRI-based realistic models, compare such knowledge with clinical findings, and define the role of computational knowledge in optimizing tsDCS protocols. tsDCS-induced electric fields are predicted to be safe and induce both transient and neuroplastic changes. This could support the possibility to explore new clinical applications, such as spinal cord injury. For the most applied protocol (2–3 mA for 20–30 min, active electrode over T10–T12 and the reference on the right shoulder), similar electric field intensities are generated in both ventral and dorsal horns of the SC at the same height. This was confirmed by human studies, in which both motor and sensitive effects were found. Lastly, electric fields are strongly dependent on anatomy and electrodes’ placement. Regardless of the montage, inter-individual hotspots of higher values of electric fields were predicted, which could change when the subjects move from a position to another (e.g., from the supine to the lateral position). These characteristics underlines the need for individualized and patient-tailored MRI-based computational models to optimize the stimulation protocol. A detailed modeling approach of the electric field distribution might contribute to optimizing stimulation protocols, tailoring electrodes’ configuration, intensities, and duration to the clinical outcome.

## 1. Introduction

The spinal cord (SC) is a complex neuroanatomical structure containing grey nuclei and neural pathways that allow communication between peripheral organs and the brain [1], segmental spinal reflexes, coordination of movements, and many other body functions [2]. Since several in vitro and animal results showed that SC is sensitive to polarizing low-intensity direct current (DC) [3,4,5], by analogy with transcranial direct current stimulation (tDCS), transcutaneous spinal direct current stimulation (tsDCS) has been introduced [6]. tsDCS non-invasively delivers weak DC (1 to 5 mA of intensity; 0.027 to 2.3 mAh/cm^2^ of charge density) through a pair of skin electrodes, with the purpose of modulating SC activity via an induced electric field (E-field). Minor adverse effects have been reported [7,8], but changes last from minutes to hours [9,10,11]. After the pivotal study of Cogiamanian et al., 2008 [9] showing that tsDCS can modulate conduction along the spinal somatosensory pathways in humans, further exploratory clinical studies have confirmed its effects on ascending and descending spinal pathways, at multiple levels [6,9,12,13], including the segmental SC [10,14,15] and cortical regions [16,17,18,19,20]. Although preliminary and limited, current knowledge discloses promising findings and suggests the clinical efficacy of tsDCS. However, standardized stimulation protocols to induce predictable effects are still lacking [21], and those used might not deliver the optimal stimulation dose [6], leading to suboptimal effects [22]. As result, it is still not clear which neurons (or cell type) are stimulated, where [23], and the duration of induced effects [22].

Similar to any electrical stimulation technique, the neurophysiological effects of tsDCS rely on the interactions between the E-field induced in the tissues and personal anatomy [24,25]. Thus, accurate knowledge about spatial distribution of the induced E-field is pivotal not only to optimize the stimulation [1,26], explore tsDCS efficacy and interpret experimental results [23], but also to assess tsDCS safety [8]. Computational modeling is a powerful tool to disclose this information [1]. This technology relies on software with a different level of complexity, depending on the clinical question and computational resources [27], but with the final aim to predict current flow in human structures during electrical stimulation. Unlike software considering simple geometries, realistic models employ specialized software and numerical solvers (i.e., finite element methods [FEM]). FEM realistic human models based on MRI are currently the most reliable simulations [27], and have also been used combined with machine learning algorithms [28]. 

In this review, we gather computational knowledge about E-field distribution in the SC during tsDCS as disclosed in MRI-based realistic models, comparing the results with clinical findings, reviewing animal results, and discussing computational contribution to future developments.

## 2. tsDCS Modeling: Methods, Limitations, and Results

The E-field generated in the SC during electrical stimulation is a function of the electrical dose, which is determined by spatial distribution (defined by shape, position, size, and electrical properties of scalp electrodes) and temporal characteristics (waveform features, duration) of the current injected [24]. Computational modeling is currently considered the standard tool for detailed knowledge on this [29,30,31,32], and the most sophisticated models use anatomical details obtained with magnetic resonance imaging (MRI) to account for anatomical characteristics [1,27,33]. Although computational models might be individualized according to a single patient’s MR anatomical images [25,34,35], so far only models averaged from healthy subjects’ high-resolution MRI have been used for tsDCS studies (see Table 1 and Table 2). Indeed, although important parameters to understand the effects of the stimulation, the E-field is not the only factor predicting physiological and behavioral effects of electrical stimulation [24,36,37]. Rather, its interaction with individual anatomy ultimately determines the biological and neurophysiological changes that occur at the neuronal and non-neuronal level [24,38]. Besides, one should consider that the biological effect of DC stimulation relies on the polarization of the cerebral tissues (charge, in Coulomb—C), which depends upon both the strength and duration of the current applied [39,40]. Therefore, the E-field intensities predicted in computational studies should not be considered as a unique predictor of stimulation effects, because even low currents applied for a sufficient amount of time exert significant biological modifications [41].

As for transcranial electrical stimulation (tES) simulations [6], tsDCS computational modeling assumes the quasi-static regime, in which induced electric potential (ϕ) is given by the quasi-static Laplace equation, and the E-field at every point of the SC tissues is obtained by means of the following relation:

E = −∇ϕ

Among the different types, realistic human models based on MRI incorporate complex tissue geometries [33] with dielectric properties assigned according to the literature [42,43]. Still, the process includes relevant caveats, related to the physical characteristics of the model, which must be chosen as a trade-off between computational facility and actual verisimilitude. Examples are the decisions on tissues’ segmentation and conductivities [6], or the numerical artifacts introduced by the staircasing error [44]. Also, protocols for tsDCS modeling come from tES computational studies [7,45], assuming the homogeneity of methodologies, mechanisms, and effects [46], but the principles of application for tES and tsDCS should be different, because the target tissues are different [46]. 

As a general statement, computational predictions have largely confirmed the role of electrode position and anatomy to determine the distribution of the E-field [24], but with remarkable differences compared with tES. For example, the position of the reference determines the distribution on the transversal section of SC [21] and the spinal region where the current density (stimulus amplitude divided by surface area of the stimulating electrode—J) is higher [21]. However, the main direction of E-fields is longitudinal along SC, regardless of the montage [21,22,26,41,46,47,48]. Indeed, from a physical point of view, the SC is assimilable to a cable-like model, with an insulating sheath (vertebral column) containing a conductive medium (SC and cerebrospinal fluid—CSF) [26]. Also, the montage creates a distinct pattern with a maximum E-field intensity approximately half-way between the two electrodes [22,23,26]. 

In their study, Pereira et al., 2018 [48] predicted that E-field values in the lumbar grey matter (GM) and white matter (WM) are probably not sufficient to modulate spinal circuitries (see Table 3) [49,50]. However, although their protocol of stimulation was not the object of further study, still similar montages have disclosed different results [22,26]. Kuck et al., 2017 [22], Fernandes et al., 2018 [26], and Bastos et al., 2016 [51] suggested maximum E-field intensities in the lumbo-sacral GM and WM theoretically able to elicit lasting plasticity effects [52], and similar to those predicted in SC with other protocols (see Table 3 and Table 4). Indeed, notwithstanding the distance of the stimulating electrode over the skin, the injected current could easily reach the SC through the intervertebral spaces [6]. These zones contain (and are covered by) connective tissue of different types, which have higher fluid content than surrounding bone and might provide a path of least resistance for current flowing, as a suture for tES [53]. Notably, models targeting C-SC [45,46,47] support the hypothesis that electrodes placed at the cervical and high thoracic level might exert similar neuromodulatory effects in the posterior cerebellum and brainstem, with the C4-cervicomental angle (C4-CMA) and C3–T3 configurations (see Table 3) even potentially able to address mechanical-related respiratory functions [46]. Also, Parazzini et al. [21] found an E-field distribution spread toward the brainstem, potentially able to modulate supraspinal activity with a different montage (i.e., with the reference over the cranial vertex) (see Table 5). However, for this protocol, clinical evidence is limited [16,54]. These results suggest the need to integrate segmental E-fields predictions with segmental presence of the cellular elements (e.g., motoneurons, interneurons, …) [55], since different metameric levels might present different cellular types, or the same cellular types but in different numbers (e.g., Renshaw cells, which are more represented in thoracic segments [56]). Another important insight from the predictions is the presence of the same hotspots in the SC regardless of the montage, with bony edges, disk intrusions in the spinal canal, CSF narrowing, and dorsal and ventral horns developing higher values of E-field [26,46,47,48]. Also, a cyclic variation reflecting vertebral body anatomy that might even influence the intervention outcome was predicted [22]. This anatomy-dependent distribution of E-fields was already reported by Fiocchi et al., 2016 [41] in child models, wherein mean E amplitude averaged over the four child models was increased by about 50% and peak level by about 60% compared with adult models, due to the anatomical differences (see Table 6). Given the theoretical value of this knowledge and the pitfalls that might bias it, computational results need to be considered and integrated with experimental studies [1].

**Table 1:** Characteristics of Duke [33], Ella [33], Billie [57], and Louis [57] models.

Duke		Ella		Billie		Louis
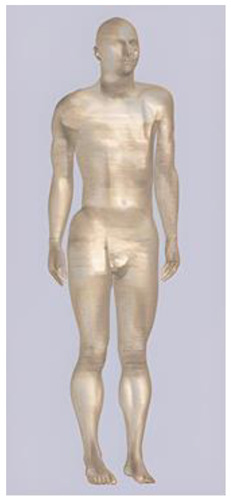		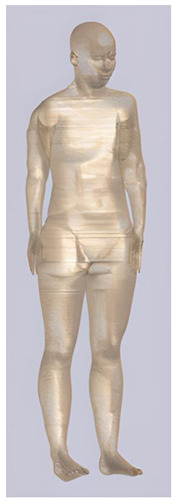		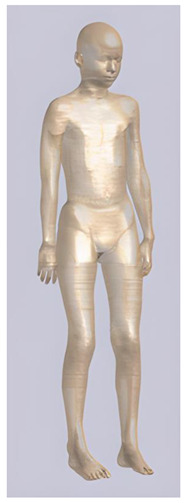		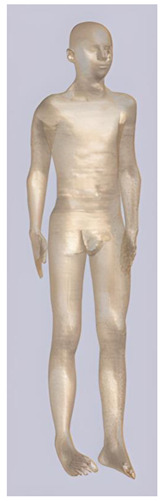
**Name**	**Age (Years)**	**Sex**	**Height (m)**	**Mass (kg)**	**BMI (kg/m^2^)**	**No. of Tissues**
Duke	34	M	1.74	70	23.1	77
Ella	26	F	1.60	58	22.7	74
Billie	11	F	1.47	35	-	75
Louis	14	M	1.69	50.4	-	77

**Table 2:** Characteristics of Roberta, Thelonious, Eartha, and Dizzy [41] models.

Roberta		Thelonious		Eartha		Dizzy
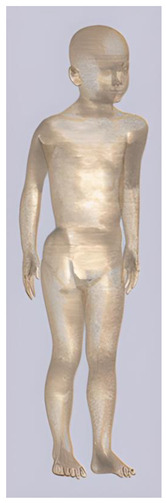		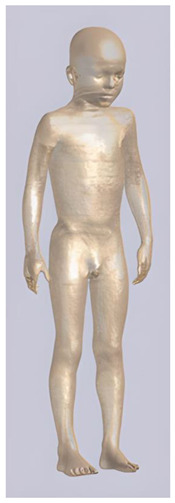		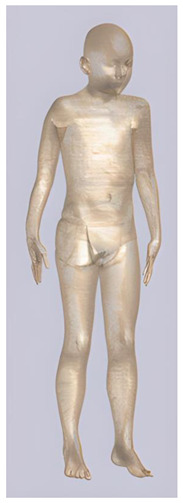		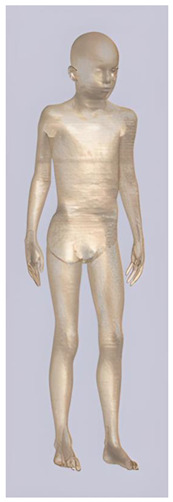
**Name**	**Age (Years)**	**Sex**	**Height (m)**	**Mass (kg)**	**BMI (kg/m^2^)**	**No. of Tissues**
Roberta	5	F	1.10	17.8	14.9	76
Thelonious	6	M	1.15	19.3	14.1	76
Eartha	8	F	1.36	30.7	16.6	76
Dizzy	8	M	1.37	26.0	13.8	76

**Table 3:** Computational results from Duke model.

Computational Studies—Duke Model							
Study	tsDCS Protocol			No. of Tissues Considered	ROIs	Induced J (A/m^2^)	Induced E (V/m)
	Active Electrode	Reference Electrode	Intensity (mA)				
Miranda et al., 2016 [23]	SP of C7 ^1^	R deltoid ^1^	2.5	9	C-SC	-	M = 0.27
Bastos et al., 2016 [51]	SP of L2, L3, L4 ^1^	4 cm above active electrode ^1^	3	8	L-SC and S/C SC	-	M = 0.29
		8 cm above active electrode ^1^				-	M = 0.39
		12 cm above active electrode ^1^				-	M = 0.47
		16 cm above active electrode ^1^				-	M = 0.57
		R deltoid ^1^				-	M = 0.35
Fernandes et al., 2016 [45]	SP of C7 ^1^	R deltoid ^1^	2.5	9	GM and WM in C-SC	-	WM: $\bar{m}$ ≅ 0.15; M = 0.27; m = 0.10 GM: $\bar{m}$ ≅ 0.13; M = 0.16; m = 0.11
	SP of C3 ^2^ *	SP of T3 ^2^ *				-	WM: $\bar{m}$ ≅ 0.39; M = 0.69; m = 0.29 GM: $\bar{m}$ ≅ 0.36; M = 0.43; m = 0.33
	SP of C3 ^2^ *	SP of T3 ^2^ *				-	WM: $\bar{m}$ ≅ 0.41; M = 0.71; m = 0.29 GM: $\bar{m}$ ≅ 0.37; M = 0.44; m = 0.34
Fernandes et al., 2018 [26]	A: SP of T10 ^3^	C: R deltoid ^3^	2.5	13	GM and WM in T-SC, L-SC, and S-SC	GM: M = 0.11–0.15	M = 0.20–0.67 (T-SC)
		C: umbilicus ^3^					M = 0.2–0.44 (L/S-SC)
		C: R iliac crest ^3^					M = 0.25–0.56 (L/S-SC)
	A: SP of T8 ^3^	C: umbilicus ^3^					M = 0.20–0.63 (lower T-SC)
		C: R iliac crest ^3^					M = 0.25–0.72 (lower T-SC)
	A: SP of L2 ^3^	C: R deltoid ^3^					M = 0.20–0.59 (T-SC and L/S-SC)
		C: SP of T8 ^3^					M = 0.25–0.76 (lower T-SC)
Pereira et al., 2018 [48]	A: between SPs of L1, L2 ^3^	C: L ASIC ^3^	2.5	13	GM and WM in L-SC	WM: M = 0.078 GM: M = 0.040	WM: M = 0.12 GM: M = 0.10
Fernandes et al., 2019 [47]	A: SP of T3 ^3^	C: SP of C3 ^3^	2.5	18	GM and WM in C-SC	-	WM: M = 0.49 GM: M = 0.44
Fernandes et al., 2019 [46]	SP of C7 ^3^	R deltoid ^3^	2.5	15	GM and WM in C-SC	-	-
	SP of C7 ^3^	cervicomental angle ^3^				-	-
	SP of C4 ^3^	cervicomental angle ^3^				-	-
	SP of C3 ^3^	SP of T3 ^3^				-	WM: M = 0.50 GM: M = 0.40

SP = spinal process, C = cervical vertebra; R = right; C-SC = cervical spinal cord; M = max; L = lumbar vertebra; L-SC = lumbar spinal cord; S/C-SC = sacral and coccygeal spinal cord; GM = grey matter; WM = white matter; $\bar{m}$ = mean; m = minimum; T = thoracic vertebra; T-SC = thoracic spinal cord; L = left; ASIC = anterior superior iliac crest; * = different geometries; ^1^ = electrode area: 35 cm^2^ and sponge area: 35 cm^2^; ^2^ = electrode area: 25 cm^2^ and sponge area: 25 cm^2^; ^3^ = electrode area: 24 cm^2^ and sponge area: 25 cm^2^.

**Table 4:** Computational results from Ella model.

Computational Studies—Ella Model							
Study	tsDCS Protocol			No. of Tissues Considered	ROIs	Induced J (A/m^2^)	Induced E (V/m)
	Active Electrode	Reference Electrode	Intensity (mA)				
Parazzini et al., 2014 [21]	SP of T10 ^1^	R deltoid ^2^	3	-	SC, CE, NRs, and muscles	C-SC: $\bar{m}$ = 8.2 × 10^−4^; M = 6.4 × 10^−3^ T-SC: $\bar{m}$ = 4.6 × 10^−3^; M = 1.4 × 10^−2^	-
		umbilicus ^2^				C-SC: $\bar{m}$ = 5.7 × 10^−5^; M = 3.2 × 10^−4^ T-SC: $\bar{m}$ = 4.1 × 10^−3^; M = 1.4 × 10^−2^	-
		head vertex ^2^				C-SC: $\bar{m}$ = 3.4 × 10^−2^; M = 8.5 × 10^−2^ T-SC: $\bar{m}$ = 9.4 × 10^−3^; M = 2.8 × 10^−2^	-
Kuck et al., 2017 [22]	SP of T11 ^3^	L posterior shoulder ^3^	2.5	-	GM and WM in L-SC	-	M = 0.47–0.82
	placed at equal distance, superior and inferior to T11 ^3^					-	
	SP of T11 ^3^	L and R ASIC ^3^				-	
Kuck et al., 2019 [58]	SP of T11 ^3^	L posterior shoulder ^3^	2.5	22	SC, soft tissues and vertebrae at representative levels (C2, T2, T6, T10)	without implants: Vertebrae: M = 0.11 (T6) Soft tissues: M = 0.55 (T6) SC: M = 0.4 (T6) with implants: Vertebrae: M = 0.11 (T6) Soft tissues: M = 0.37 (T16) SC: M = 0.11 (T6)	without implants: Vertebrae: M = 7.02 (T6) Soft tissues: M = 4.94 (T2) SC: M = 2.6 (T6) with implants: Vertebrae: M = 5.57 (T6) Soft tissues: M = 2.32 (T6) SC: M = 0.15 (T6)
	Placed 7 cm superior and inferior to T11 ^3^					without implants: Vertebrae: M = 0.15 (T6) Soft tissues: M = 0.66 (T10) SC: M = 0.59 (T10) with implants: Vertebrae: M = 0.23 (T10) Soft tissues: M = 1.04 (T10) SC: M = N.R.	without implants: Vertebrae: M = 7.7 (T6) Soft tissues: M = 5.38 (T10) SC: M = 3.6 (T10) with implants: Vertebrae: M = 11.78 (T10) Soft tissues: M = 6.52 (T10) SC: M = N.R.

SP = spinal process; T = thoracic vertebra; R = right; SC = spinal cord, CE = cauda equina; NRs = nerves roots; C-SC = cervical spinal cord; T-SC = thoracic spinal cord; $\bar{m}$ = mean; M = maximum; L = left; ASIC = anterior superior iliac crest; GM = grey matter; WM = white matter; L-SC = lumbar spinal cord; ^1^ = electrode area: 37.5 cm^2^ and sponge area: 56 cm^2^; ^2^ = electrode area: 47.5 cm^2^ and sponge area: 70 cm^2^; ^3^ = electrode area: 35 cm^2^ and sponge area: 35 cm^2^.

**Table 5:** Computational results from Billie and Louis models.

Computational Studies—Billie and Louis Models						
Study	Model	tsDCS Protocol			ROIs	Induced J (A/m^2^)
		Active Electrode	Reference Electrode	Intensity (mA)		
Parazzini et al., 2014 [21]	Louis	SP of T10 ^1^	R deltoid ^2^	3	SC, CE, NRs, and muscles	C-SC: $\bar{m}$ = 3.6 × 10^−4^; M = 2.4 × 10^−3^ T-SC: $\bar{m}$ = 5.4 × 10^−3^; M = 1.6 × 10^−2^ L-SC: $\bar{m}$ = 3.9 × 10^−3^; M = 6.1 × 10^−3^
			umbilicus ^2^			C-SC: $\bar{m}$ = 4.7 × 10^−5^; M = 4.1 × 10^−4^ T-SC: $\bar{m}$ = 4.9 × 10^−3^; M = 1.6 × 10^−2^ L-SC: $\bar{m}$ = 1.2 × 10^−2^; M = 1.7 × 10^−2^
			head vertex ^2^			C-SC: $\bar{m}$ = 3.4 × 10^−2^; M = 7.9 × 10^−2^ T-SC: $\bar{m}$ = 1.6 × 10^−2^; M = 3.3 × 10^−2^ L-SC: $\bar{m}$ = 3.8 × 10^−3^; M = 6.0 × 10^−3^
	Billie	SP of T10 ^1^	R deltoid ^2^			C-SC: $\bar{m}$ = 6.5 × 10^−4^; M = 3.4 × 10^−3^ T-SC: $\bar{m}$ = 6.3 × 10^−3^; M = 1.4 × 10^−2^ L-SC: $\bar{m}$ = 2.3 × 10^−3^; M = 1.1 × 10^−2^ S/C-SC: $\bar{m}$ = 9.2 × 10^−4^; M = 1.7 × 10^−3^
			umbilicus ^2^			C-SC: $\bar{m}$ = 1.5 × 10^−4^; M = 5.2 × 10^−4^ T-SC: $\bar{m}$ = 5.8 × 10^−3^; M = 1.9 × 10^−2^ L-SC: $\bar{m}$ = 1.0 × 10^−2^; M = 2.4 × 10^−2^ S/C-SC: $\bar{m}$ = 2.3 × 10^−3^; M = 4.3 × 10^−3^
			head vertex ^2^			C-SC: $\bar{m}$ = 4.0 × 10^−2^; M = 6.3 × 10^−2^ T-SC: $\bar{m}$ = 1.4 × 10^−2^; M = 3.2 × 10^−2^ L-SC: $\bar{m}$ = 1.6 × 10^−3^; M = 8.5 × 10^−3^ S/C-SC: $\bar{m}$ = 2.5 × 10^−4^; M = 4.6 × 10^−4^

SP = spinal process; T = thoracic vertebra; R = right; SC = spinal cord; CE = cauda equina; NRs = nerves roots; C-SC = cervical spinal cord; T-SC = thoracic spinal cord; L-SC = lumbar spinal cord; S/C-SC = sacral and coccygeal spinal cord; $\bar{m}$ = mean; M = max; ^1^ = electrode area: 37.5 cm^2^ and sponge area: 56 cm^2^; ^2^ = electrode area: 47.5 cm^2^ and sponge area: 70 cm^2^.

**Table 6:** Computational results from Roberta, Thelonious, Eartha, and Dizzy models.

Computational Studies—Roberta, Thelonious, Eartha and Dizzy Models						
Study	Model	tsDCS Protocol			ROIs	Induced E (V/m)
		Active Electrode	Reference Electrode	Intensity (mA)		
Fiocchi et al., 2016 [41]	Roberta	SP of T10 ^1^	R deltoid ^2^	3	SC, CE and NRs	C-SC: me ≅ 0.1; M ≅ 0.5; m ≅ 0.01 T-SC: me ≅ 1.6; M ≅ 2.7; m ≅ 0.3 L-SC: me ≅ 0.8; M ≅ 1.2; m ≅ 0.45
	Thelonious	SP of T10 ^1^	R deltoid ^2^			C-SC: me ≅ 0.1; M ≅ 0.5; m ≅ 0.01 T-SC: me ≅ 1.6; M ≅ 2.7; m ≅ 0.3 L-SC: me ≅ 0.8; M ≅ 1.2; m ≅ 0.45
	Eartha	SP of T10 ^1^	R deltoid ^2^			C-SC: me ≅ 0.15; M ≅ 0.25; m ≅ 0.01 T-SC: me ≅ 0.6; M ≅ 1.8; m ≅ 0.25 L-SC: me ≅ 0.25; M ≅ 0.3; m ≅ 0.15
	Dizzy	SP of T10 ^1^	R deltoid ^2^			C-SC: me ≅ 0.1; M ≅ 0.25; m ≅ 0.01 T-SC: me ≅ 0.8; M ≅ 1.6; m ≅ 0.2 L-SC: me ≅ 0.25; M ≅ 0.4; m ≅ 0.15

SP = spinal process; T = thoracic vertebra; R = right; SC = spinal cord, CE = cauda equina; NRs = nerves roots; C-SC = cervical spinal cord; T-SC = thoracic spinal cord; L-SC = lumbar spinal cord; me = median; M = maximum; m = minimum; ^1^ = electrode area: 15 cm^2^ and sponge area: 15 cm^2^; ^2^ = electrode area: 25 cm^2^ and sponge area: 25 cm^2^.

## 3. Computational Insights for Clinical Studies

In most of the clinical studies [15,59,60,61,62,63,64,65,66,67,68,69,70], tsDCS has been applied with a similar protocol, i.e., at 2–3 mA for 20–30 min, with the active electrode over the lower part of the thoracic spine (T10–T12) and reference on the right shoulder (anterior, lateral, or posterior zone). For this protocol, computational simulations reported no harmful effect in SC [21,22], or in the presence of metallic spinal implants [58]. For example, Kuck et al. [22] obtained E-field values of more than a thousandfold lower than the safety limits for tissue damage [71,72]. Likewise, no serious adverse effects have been clinically reported in human studies so far, nor blood biomarkers indicative of neuronal damages were detected immediately after stimulation offset [9]. As for E-field distribution induced by these protocols, the stimulation produces mainly a longitudinal E-field along almost all the vertebral column [21,22,26], and in particular in the regions between the electrodes [22], with a ratio between longitudinal and transverse components of the field of mean value ranging from 3 (for spinal-WM) and 6 (for spinal-GM) [21,26]. Since several findings suggest that longitudinal E-fields induced by SC stimulation might have a prominent role in promoting axonal regrowth and/or preventing degeneration [73], these observations might support the potential role of tsDCS in SC-injured patients. tsDCS induced E-fields are on average 10 times lower than those expected to have a biological effect [21,26,74,75]; however, cellular responses have been demonstrated to be dependent on the duration [76,77] and intensity of the stimulation [78,79], meaning that even weak current applied for a long time might exert a biological effect. For example, morphological neuronal changes (axonal outgrowth and regeneration) were reported in a guinea pig model of SCI after stimulation intensity lower than that used in human tDCS but for a longer duration [80]. Notably, axonal regeneration was also found across a scar on the SC [80]. tsDCS is expected to elicit only spinal circuitry neuromodulation via efferent axon polarization at the terminal [26]. However, the electrical dose and outcome relationship is still not well characterized in tsDCS, but following tES studies it is reasonable to presume that it may follow a non-linear relation; thus, higher E-fields intensity may not necessarily lead to an increase of effects [81]. Besides longitudinal pathways, tsDCS may modulate spinal reflexes and interneuronal spinal networks, acting also within spinal levels. Anodal tsDCS increases the efficacy of the Ia–motoneuron synapse [10] and induces a leftward shift of the soleus H-reflex recruitment curve [14]; cathodal tsDCS reduces the efficacy of the Ia fibre–motoneuron synapse [10] and spinal reflex amplitudes in healthy subjects [15]. Still, E-fields developed during the clinically applied tsDCS protocols might be able to elicit lasting plasticity effects [26,52,57]. This prediction integrates the results from clinical studies suggesting that the tsDCS effect might go beyond a polarization of neuron membrane alone and involve neuroplasticity [82,83]. Indeed, the reduction of lower limb flexion reflex amplitude in healthy subjects [12] and improvement of motor behavior in subjects with primary orthostatic tremor [61] lasted for more than 30 min after the current offset. Likewise, beneficial effects on severity of restless legs syndrome and sleep performances were found by Zeng et al. up to 2 weeks after the treatment with anodal tsDCS [84]. 

For the active electrode thoracic spine and reference over the right shoulder, similar E-field intensities are generated in both ventral and dorsal horns of the SC at the same height [21,26]. This occurs between the electrodes [21,26], with maximum E-field intensity in WM and GM located in the tract T5–T12 [26]. This means that tsDCS might exert a non-selective neuromodulatory effect in spinal sensory (dorsal) and motor (ventral) nuclei, which is in line with several clinical studies [9,12,13,19]. For example, in healthy subjects, anodal tsDCS inhibits dorsal column pathways, reducing amplitudes of tibial nerve somatosensory-evoked potentials (SEPs) [9,12], and spinothalamic nociceptive pathways, reducing the amplitudes of laser-evoked potentials [13]; also, it decreases the excitability of the entire corticospinal tract, as evidenced by the increase in resting motor threshold in abductor hallucis [19]. This phenomenon is reported as conceptually similar to the “anodal block” [7,85]. A similar inhibitory effect was found for multiple sclerosis patients with central neuropathic pain [63], and patients with chronic headache [60], primary orthostatic tremor [61], and restless legs syndrome [59,62]. In patients, anodal tsDCS possibly modulated anterior grey columns, reducing spasticity in patients with hereditary spastic paraplegia [68]; instead, cathodal tsDCS failed to change the Ashworth scale of the affected lower limb in patients with chronic stroke [70]. 

In the study by Wang et al., 2020, neuroimaging techniques (MRI and resting-state fMRI) suggested a cortical effect of anodal tsDCS with active electrode over T10 and reference over the right shoulder (e.g., decrease in cortical GM volume in bilateral cuneus and left post central gyrus and increase in functional connectivity between bilateral cuneus and left primary visual cortex, right cuneus, and right lingual gyrus) [59]. Several findings suggest a tsDCS-induced modulation, possibly due to an indirect effect, of higher anatomical structures, with supraspinal effects both in healthy subjects [17,18,19,20,86] and in patients with post-stroke aphasia [64,65], incomplete SC injury [67], and Alzheimer’s disease [66]. Also, tsDCS (regardless of the polarity) may affect transcallosal processing [18,87], as reported for other types of spinal stimulation [88,89]. However, no computational models have explored this aspect so far for the stimulation protocol considered.

## 4. Insights for Clinical Studies from Animal Models

Recently, several studies have investigated tsDCS effects on animal models, providing a biological framework for tsDCS mechanisms, applications, and methodologies [6]. For example, the use of computational and experimental techniques helped Williams et al., 2022 [55] to characterize tsDCS-induced neuromodulation in a cat model, providing an anatomical substrate to the neurophysiological results. The authors studied the effects of current intensity (1, 2, 3, 4, 5 mA), polarity, and electrode position (active electrode over C2–C6 or T2–T6, reference over sternal manubrium) on proximal and distal forelimb muscle activation. Cathodal and anodal current modulated, respectively, enhancement and suppression of motor evoked potentials (MEP). More importantly, the authors were able to define that the cathode location effective in modulating MEPs of proximal muscles, steered more current rostrally in the cervical cord; conversely, the location effective for distal muscles induced more current caudally [55].

In rats, tsDCS induced polarity-specific effects [3,4,90]. Anodal increased single-unit activity in SEPs (1 mA for 15 min, active electrode over thoracic SC, reference over abdominal area) [90], but depressed twitch force (for triceps surae) and increased latency (tibial nerve) in motor-evoked potentials (MEPs) (0.5 to 3 mA for 3 min, Active electrode at T10–L1, and reference on lateral abdominal muscles) [3]. Cathodal tsDCS exerted opposite effects [3,90], but increased ankle and multi-joint movements elicited through cortical stimulation (0.8 mA for 8 sec, active electrode on lumbar enlargement area, reference on abdominal skin flap) in another study on anaesthetized mice [4]. From a translational point of view, animal models have been used to explore tsDCS for SC injury (SCI) treatment [91,92,93,94]. Preclinical studies have mainly focused on lower urinary tract function [91] and motor recovery [92,93,94]. Cathodal tsDCS was able to modulate urinary functions [91], with 1 mA DC applied for 20 min (active electrode over area from the L2 to the L5 vertebral level, reference over lateral abdominal skin) changing bladder and external urinary sphincter reflexes in mice with severe contusive SCI and overactive bladder [91]. Promising results were also reached for motor symptoms in SCI. Ahmed et al., 2013 [92] found that cathodal tsDCS together with cortico-sciatic stimulation or repetitive cortical electrical stimulation improved walking recovery in unilateral SCI animals. Also, combined with bilateral intermittent theta burst stimulation (iTBS) of motor cortex, cathodal tsDCS (1.5 mA, active electrode over the C4–T2 vertebrae, reference over the chest) significantly promoted axonal sprouting in the corticospinal tract below and above the level SC contusion, with recovery of skilled locomotion and forepaw manipulation skills, in injured rats [93]. This same protocol was replicated by Yang et al., 2019 [94], with similar results; below the lesion, the axon length was double that of the control rats, suggesting an association of movement recovery and neuronal sprouting [94]. Spasticity, which is another frequent motor symptom that gradually onsets over several months after injury [95], has been targeted for tsDCS treatment. Mouse models receiving 1.5 mA tsDCS for 20 min, once a day for 7 days (anode over the dorsum of the SC, cathode over abdominal skin) had significant reductions in spasticity, ground locomotion, and skill locomotion [96]. Other authors have used animals to investigate biochemical underpinning of tsDCS [3,92]. In mice, the mechanisms of cathodal DC stimulation might involve GABA and glycine receptors [3], and glutamate metabolism [97]. However, studies in animals have some limitations. Heterogeneity in stimulation protocols [6] and the experimental methodologies [98,99], together with the characteristic anatomy of the animals, might affect the results.

## 5. What Is Next? Role of Models for tsDCS Applications

Since the evidence indicates that the target location should guide the choice of an appropriate electrode montage, and that the E-fields developed in SC are low enough to be safe, but high enough to induce both transient and neuroplastic changes, modeling predictions may guide future tsDCS clinical applications. The evidence that personal anatomy strongly affects E-fields, for example, is of particular interest in the clinical setting and urges the need for personalized protocols, rather than just replicating historical modulatory parameters. The presence of anatomical changes such as protrusions of herniated disks (or any other mass) in the spinal canal [22,26] needs to be carefully considered in planning and performing the stimulation. For example, Kuck et al. [58] simulated the presence of spinal metallic implants during tsDCS (see Table 4). At least in theory, the implant may lead to an altered current flow, increasing the chance of locally high current concentrations with potential damaging effects. Although no studies have specifically assessed the tsDCS dose, animal studies in combination with computational simulations suggest J as a predictor for DC stimulation-evoked tissue damages [72], and estimate that J of 6.3 A/m^2^ to 17 A/m^2^ might be harmful [7,72,100]. In the study, J was predicted to be below these magnitudes [58]. The inter-individual variabilities must be considered also in terms of electrode placement, since finding the correct vertebrae can sometimes be difficult [22], depending on subject anatomy or position. However, when the active electrode is misplaced, E-field intensity and direction at the stimulation target site are altered, with a potentially large effect on axon terminal polarization [22]. Computational simulations suggest that EF distribution seems to be remarkably sensitive to electrode misplacement, with longitudinal offset of even 5 cm (ca. 1.5 vertebra length) sufficient to induce significant magnitude changes [22]. Since each spinal segment contains neuron circuitry related to specific functions [26], electrode misplacement must be considered during clinical application of tsDCS. Also, moving from one position to another (e.g., from the supine to the lateral position) induces anterior shift of the SC center of mass and associated nerve roots [101], with CSF narrowing in different SC regions and changes in the location of E-field hotspots. The subject position during tsDCS might be a source of variability in experimental studies, but also a way to enhance neuromodulation effects over a target region if adequately selected. All this information coming from computational predictions needs to be carefully considered for the next experimental tsDCS studies to reach that methodological homogeneity necessary to move the technique to clinical practice. Indeed, several aspects of tsDCS are still not well understood, and computational predictions could help in characterizing them–for example, the relationship between stimulation time and modulation polarity. Recently, a non-linear relation has been described in tDCS, where anodal stimulation over the primary cortex starts to shift from excitatory to inhibitory after 26 min of stimulation [102]. A possible example for this may be found in Awosika et al. [69], who assessed the effects of tsDCS combined with backward locomotion treadmill training in the walking capacity of patients with chronic stroke. The active electrode (anode) was centered over the 11th thoracic spinous process and the return electrode over the right shoulder. Current intensity was set at 2.5 mA for 30 min. Despite employing parameters that have demonstrated effects in corticospinal excitability [17,19], there were no significant differences between the anodal and sham group, but a tendency of sham to be better than the active group. Considering that most tsDCS clinical trials have applied a modulation time of 20 min, it is not possible to exclude a potential polarity shift due to time [69]. Another unclear aspect to be addressed for tsDCS optimization (and potential source of variability for clinical studies) is the role of the basal state of the target area [103]. It is plausible that results obtained in healthy subjects might not be reproducible in clinical populations. TsDCS in healthy subjects have shown to modulate spinal conduction characteristics [17,19,102]; nevertheless, clinical studies have presented inconsistent results. Contrary to the reports of Bocci et al. [17,19] and Winkler et al. [10] in normal subjects, Ardolino et al. [68] failed to find an effect in motor evoked potentials and H-reflex after anodal tsDCS in patients with hereditary spastic paraplegia possibly due to a progressive loss of corticospinal fibres [68]. Further, implementing concurrent tasks or interventions may also modify the ongoing neural activity and affect tsDCS effects [103]. Paget-Blanc et al. [104] reported significant improvements in upper limb motor function and spasticity of stroke patients after five days of tsDCS paired with peripheral DC stimulation. Whereas Picelli et al. found a significant improvement in walking distance, but not in spasticity or limb motricity after anodal tDCS paired with cathodal tsDCS [70]. In addition, the potential influence of different genotypes in spinal plasticity should be taken into consideration. Among them, brain-derived neurotrophic factor (BDNF) Val66Met polymorphism has been suggested to be associated with altered motor cortex plasticity [105,106]. Similar to previous non-invasive brain stimulation studies [107,108,109], Lamy et al. detected a different neuroplastic effect in healthy subjects carrying the BDNF Val66Met polymorphism compared to age and sex matched valine homozygotes after anodal tsDCS [110]. While the Val/Val carriers presented a left shift in the recruitment curve of the H reflex, Met allele carriers remained reluctant to the stimulation [110]. Because further influences of BDNF polymorphisms or other plasticity-related genes are unknown, future studies should consider genotyping as part of their protocols to control potential sources of inter-individual variability and identify clusters of responders to DC. 

Overall, tsDCS computational modeling might help researchers to explore in a more confident and reliable manner different protocols, including current intensities, densities, durations, and repetitions required for a desired long-lasting effect [47].

## 6. Concluding Remarks

tsDCS is a promising non-invasive neuromodulation technique that needs to be better understood and studied. As for tES, computational studies on tsDCS have been used to characterize the electrical effects of the stimulation inside human tissues [111]. Here, we reviewed the current computational knowledge about tsDCS, and compared it with human findings. Although this approach might present some limitations, e.g., matching results coming from different populations (high-resolution MRI of healthy volunteers for computational studies, patients for human studies) and with different grades of clinical quality, it might boost the role of computational models in optimizing tsDCS delivery. Besides confirming its safety, predictions disclosed that the induced E-field is dependent on the montage and the personal anatomy. Also, E-fields potentially able to induce neuroplastic effects were predicted. However, many other aspects of the stimulation, and of its interaction with neurophysiology, have not been completely understood. This might partially explain the heterogeneity of actual results. In this scenario, future research directions may include: (I) the use of a detailed modeling approach to personalize the stimulation, for example relying on models based on individual MRI to predict electric current distribution in each subject, as already proposed for other stimulation techniques [34,35]. This would allow researchers to better identify the mechanisms of action underlying the after-effects of spinal polarization, thus contributing to improving the treatment in terms of electrodes’ configuration, intensities, and duration. Also, technological implementations (e.g., algorithms of artificial intelligence) might offer great opportunities for other forms of electrical stimulation [28], and neuromorphic computers might enhance computational resources [112] to be exploited to characterize, among the others, neurons and synapses behavior and neuroplasticity [113]; (II) the use of a detailed modeling approach to study the effects of DC over molecular outcomes rather than just clinical or neurophysiological, for example, in SCI in acute stages, to limit the neuronal degeneration and/or promote the recovery. Previous in vitro studies suggest that DC may aid in the clearance of pathological intracellular proteins related to neurodegeneration [114,115]. Besides, in vivo studies in rodents have shown a potential anti-inflammatory effect of weak currents [115,116,117]. If confirmed in humans, these results may open new therapeutic possibilities for neurodegenerative disease [118], which are characterized by neuroinflammation [119] and accumulation of aberrant proteins [120]. Also, several in vitro and in vivo results suggest that tissue polarization induced by exogenous E-fields might promote neuronal regeneration and axonal sprouting [115]; (III) the confirmation of modeling results by studies with a more solid methodology in terms of larger sample sizes, more homogenous population, and optimized study design (double-blinded, parallel design).

## Data Availability

Not applicable.
